# Left Ventricular Global Longitudinal Strain as a Marker of Subclinical Myocardial Dysfunction in Youths With Type 1 Diabetes Mellitus: An Updated Systematic Review and Meta‐Analysis

**DOI:** 10.1002/dmrr.70220

**Published:** 2026-08-25

**Authors:** Athina Stamati, Georgia Sotiriou, Elpida Emmanoulidou‐Fotoulaki, Nikoleta Printza, Maria Kavga, Athanasios Christoforidis

**Affiliations:** ^1^ Faculty of Health Science School of Medicine Aristotle University of Thessaloniki Thessaloniki Greece; ^2^ 1st Paediatric Department Faculty of Health Sciences School of Medicine Aristotle University of Thessaloniki Ippokratio General Hospital Thessaloniki Greece; ^3^ 3rd Paediatric Department Faculty of Health Sciences School of Medicine Aristotle University of Thessaloniki Ippokratio General Hospital Thessaloniki Greece

**Keywords:** global longitudinal strain, meta‐analysis, myocardial dysfunction, systematic review, type 1 diabetes mellitus

## Abstract

**Aims:**

To assess Left Ventricular Global Longitudinal Strain (LV GLS) in youths with type 1 diabetes mellitus (T1DM) compared with healthy controls, as a marker of subclinical myocardial dysfunction.

**Methods:**

We searched MEDLINE, the Cochrane Library, Scopus and the Web of Science up to November 2025 for studies comparing speckle‐tracking‐derived LV GLS in paediatric T1DM and healthy controls. Random‐effects meta‐analyses focused on two‐dimensional LV GLS, with exploratory three‐dimensional analyses, vendor‐based subgroup analyses and meta‐regression for age, diabetes duration and HbA1c.

**Results:**

Twenty studies met the inclusion criteria. Eighteen studies (1086 youths with T1DM, 935 controls) were included in the primary 2D LV GLS analysis. Compared with controls, youth with T1DM had significantly impaired 2D LV GLS (mean difference −2.87%, 95% CI −4.03 to −1.71), with substantial heterogeneity (I^2^ = 96.2%). Exploratory 3D GLS analysis with three studies showed no significant between‐group differences, and meta‐regression revealed no associations with age, diabetes duration or HbA1c.

**Conclusion:**

Children and adolescents with T1DM show impaired left ventricular longitudinal deformation despite preserved conventional systolic indices, indicating early subclinical myocardial dysfunction. However, given the substantial heterogeneity and low certainty of current evidence, future standardized longitudinal studies are warranted. Ultimately, with the increasing use of speckle‐tracking echocardiography, GLS may serve as a valuable tool for cardiovascular risk stratification in this population.

## Graphical abstract

Children and adolescents with type 1 diabetes show significantly impaired left ventricular global longitudinal strain compared with healthy controls, indicating subclinical myocardial dysfunction. Echocardiographic strain imaging detects early myocardial alterations in paediatric T1DM and may support timely cardiometabolic risk management.

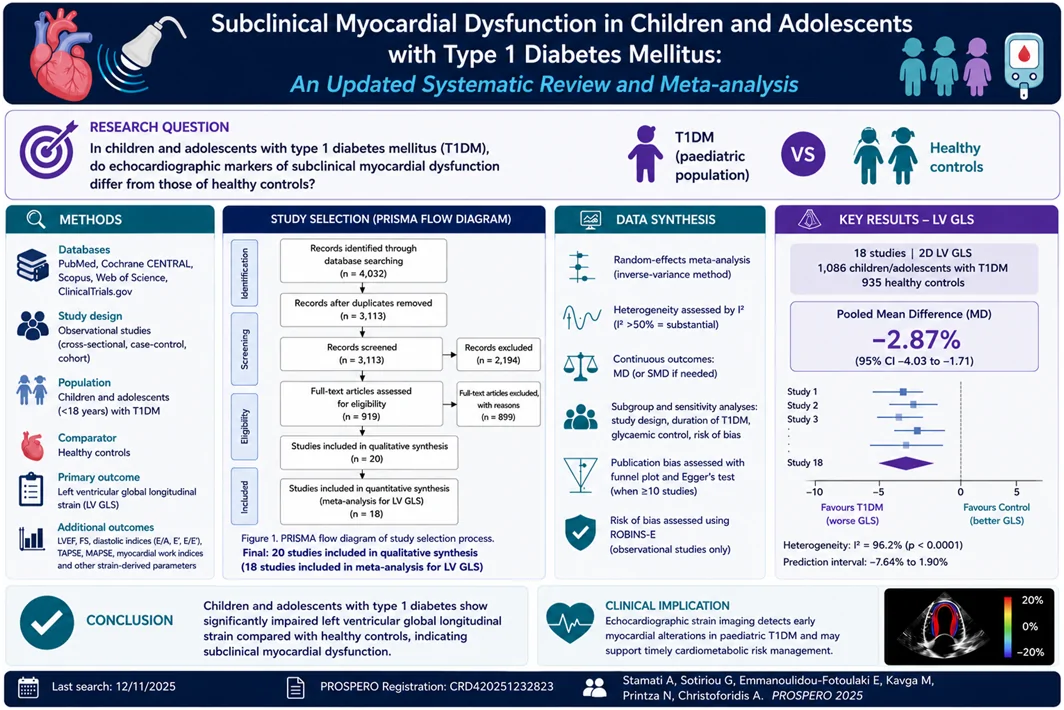

## 1 Introduction

Type 1 diabetes mellitus (T1DM) is a chronic, immune‐mediated disorder that most often occurs in childhood and adolescence, characterised by autoimmune destruction of pancreatic β‐cells and near absolute insulin deficiency [1]. T1DM remains the predominant form of diabetes in youth [2], with a rising global incidence and an annual increment of 3%–5% particularly among younger age groups [3]. Given this growing burden, the presence of T1DM in childhood is not only a public health challenge, but also a critical period where early, even subclinical, organ damage may already begin to develop, long before overt complications become clinically apparent.

Subclinical myocardial dysfunction in children and adolescents with T1DM represents early impairment of cardiac performance that precedes symptomatic heart disease and can exist despite otherwise unremarkable clinical examinations and routine cardiac indices [4]. These alterations typically involve subtle impairment of both systolic and diastolic myocardial function, reflecting early compromise of myocardial contractility and relaxation rather than advanced structural heart disease [5]. Conventional measures frequently remain within normal limits, allowing myocardial involvement to go undetected in routine clinical practice [6]. Paediatric patients with T1DM exhibit measurable alterations in myocardial deformation compared with healthy controls—including reduced longitudinal strain and diastolic abnormalities—despite preserved conventional systolic indices [5, 7, 8]. Accumulating evidence links this subclinical impairment to disease duration, cumulative glycaemic exposure and microvascular complications, supporting a pathophysiological continuum from metabolic stress to early myocardial injury [9]. Early recognition and characterisation of this cardiac involvement during childhood may therefore be crucial for improving mechanistic understanding and for identifying children with T1DM who may be at higher cardiovascular risk and could potentially benefit from targeted surveillance and preventive strategies.

Two‐dimensional speckle‐tracking echocardiography (2D‐STE) has increasingly been recognized as a sensitive and reproducible echocardiographic modality for the detection of subclinical myocardial dysfunction in adults, providing quantitative assessment of myocardial deformation beyond conventional parameters such as left ventricular ejection fraction (LVEF) [8]. Unlike standard echocardiographic measures that often remain normal in early stages of disease, speckle‐tracking allows calculation of Global Longitudinal Strain (GLS), a metric reflecting the percentage of myocardial fibre shortening along the long axis and thereby early systolic performance [10, 11]. A preceding systematic review and meta‐analysis by Gupta et al. demonstrated significantly reduced left ventricular GLS (LV GLS) in children and adolescents with T1DM compared with healthy controls, despite preserved conventional systolic measures [12]. However, since its publication, the evidence base has substantially expanded. Building on this previous work, the present meta‐analysis incorporates a significantly larger and more contemporary dataset, analysing 20 studies comprising 1188 T1DM patients and 1028 controls, aiming to provide an updated quantitative synthesis. Furthermore, important sources of methodological and clinical heterogeneity were left unaddressed in prior reviews. The present study addresses these specific gaps by implementing meta‐regression models to evaluate the impact of clinical covariates, specifically age, diabetes duration and cumulative glycaemic exposure (HbA1c). In addition, we perfectly align our analytical framework with the stated methodological gaps by systematically exploring vendor‐specific software variability through prespecified subgroup analyses—an aspect not previously examined in paediatric populations—and by conducting a separate exploratory quantitative synthesis of three‐dimensional (3D) speckle‐tracking data. Ultimately, this updated review is warranted to address the growing clinical adoption of speckle‐tracking echocardiography and to clarify the methodological and clinical factors influencing LV GLS estimates, enhancing its clinical applicability for cardiovascular risk stratification in paediatric T1DM.

## 2 Material and Methods

This systematic review and meta‐analysis was conducted in accordance with the Preferred Reporting Items for Systematic Reviews and Meta‐Analyses (PRISMA) statement. The study protocol was prospectively registered in the PROSPERO international database (registration number CRD420251232823).

### 2.1 Data Sources

A comprehensive and systematic literature search was conducted in MEDLINE (via PubMed), Cochrane Central Register of Controlled Trials (CENTRAL), Scopus and Web of Science up until November 2025. In addition, trial registries, including ClinicalTrials.gov, were manually searched to identify ongoing or unpublished studies. To ensure completeness, reference lists of all eligible articles and relevant reviews were also screened for additional studies. Conference proceedings from major international scientific meetings, including those of the American Diabetes Association (ADA), the European Association for the Study of Diabetes (EASD) and the European Society for Paediatric Endocrinology (ESPE) were reviewed for potentially eligible abstracts. If full‐text articles were not accessible through electronic databases or institutional subscriptions, corresponding authors were contacted via email a maximum of two times, with a two‐week interval between each attempt, to request additional information or unpublished data. The search strategy combined Medical Subject Headings (MeSH) and free‐text terms related to type 1 diabetes mellitus, paediatric population, echocardiography and myocardial dysfunction. The complete search strategy for each database is provided in the Supporting Information S1: Table S1.

### 2.2 Study Selection

All identified records from the database searches and additional sources were imported into reference management software (Mendeley) and duplicates were removed. Titles and abstracts were independently screened by two reviewers (A.S. and E.E.‐F.) to assess relevance based on the pre‐specified inclusion criteria. Full texts of potentially eligible studies were then retrieved and evaluated in detail. Discrepancies at any stage of screening were resolved through discussion, and, if necessary, a third reviewer (A.C.) was consulted to reach consensus. Studies were included if they reported LV GLS in children and adolescents with T1DM, either compared with healthy controls or with extractable normative data. Both observational studies and randomized controlled trials (RCTs) reporting baseline or independent control arm data were considered eligible, while studies without paediatric‐specific results, case reports, reviews, conference abstracts lacking sufficient data and animal studies were excluded.

### 2.3 Data Extraction

Data extraction was conducted independently by two reviewers (A.S. and E.E.‐F.) using a pre‐designed Microsoft Excel form to ensure standardized and consistent recording of study information. Extracted data included study characteristics, participant demographics, T1DM‐related characteristics and LV GLS values for both T1DM and healthy control groups. The primary outcome of interest was LV GLS (%) as a marker of subclinical myocardial dysfunction in children and adolescents with T1DM. When studies reported LV GLS using different sign conventions, values were harmonised prior to pooling by aligning the negative sign convention for myocardial shortening to ensure directional comparability across studies. No unit‐scaling multiplication factors were required as all parameters were expressed in percentages (%). In cases where systematic differences in measurement methods remained, standardized mean differences (SMD) were calculated for meta‐analysis. For studies reporting multiple nonindependent comparisons within a single publication, data were handled according to Cochrane guidelines to avoid double‐counting, either by combining relevant arms into a single summary estimate or by including only the most appropriate comparison [13]. When measures of variability such as standard deviation (SD), standard error (SE), or 95% confidence intervals (CI) were not reported, SD was calculated from *p*‐values, or, if unavailable, SD was estimated using data from comparable studies with similar population characteristics [14, 15].

### 2.4 Risk of Bias Assessment

For non‐randomized observational studies, the ROBINS‐I V2 tool was used to assess risk of bias across six domains, including confounding, participant selection, classification of exposures, missing data, measurement of outcomes and selection of the reported result [16, 17]. The assessment was conducted in an outcome‐specific manner for the primary outcome (LV GLS). A priori baseline confounders were prespecified based on biological plausibility and prior paediatric echocardiography literature, and included age, sex, duration of type 1 diabetes and glycaemic control (HbA1c) [18, 19]. These confounders are routinely reported and considered clinically relevant in paediatric studies assessing myocardial deformation. The expected direction of bias for each confounder, if unadjusted, was considered as part of the confounding domain assessment. Each domain was judged as being at low, moderate, serious or critical risk of bias according to the ROBINS‐I v2 tool. The overall risk of bias for each study was determined by the highest risk attributed to any individual domain. Studies judged to be at a serious risk of bias were not excluded from the primary analysis but were explored in sensitivity analyses to assess the robustness of pooled estimates. Risk of bias assessments were performed independently by two reviewers (A.S. and E.E.‐F.), with disagreements resolved through discussion and, when necessary, consultation with a third reviewer (A.C.).

### 2.5 Data Synthesis

Statistical analyses were performed using the R Project for Statistical Computing (Version 4.5.2). The primary outcome, LV GLS, was analysed as a continuous variable and mean differences (MD) with 95% confidence intervals (CI) were calculated. LV GLS was eligible for inclusion whether assessed by two‐dimensional (2D) or 3D speckle‐tracking echocardiography, in accordance with the study protocol. To make full use of the evidence base, an initial overall combined analysis of both 2D and 3D studies was performed. Because 2D and 3D imaging represent distinct modalities with inherent differences in spatial and temporal resolution, this combined synthesis was conducted using SMD. Subsequently, given the substantially wider use of 2D GLS and its greater methodological consistency, 2D GLS was prioritised for the primary absolute MD quantitative synthesis. In studies reporting both 2D and 3D GLS, only 2D GLS estimates were included in the primary 2D meta‐analysis to avoid double counting. Studies reporting LV GLS using 3D speckle‐tracking echocardiography were also synthesised separately in an exploratory meta‐analysis.

A random‐effects meta‐analysis using the inverse‐variance method was conducted to account for variability across studies. Heterogeneity was quantified with the I^2^ statistic, with I^2^ > 60% considered indicative of substantial heterogeneity [15]. Sensitivity analyses were conducted by excluding studies at serious or critical risk of bias and subgroup analyses were conducted based on the software used. Subgroup differences were considered statistically significant if the p‐interaction value was ≤ 0.05. Publication bias was evaluated with funnel plots and Egger's regression test if ≥ 10 studies contributed to the pooled outcome. All effect estimates are reported with 95% CIs and statistical significance was defined as *p* < 0.05.

Meta‐regression analyses were conducted to investigate whether selected study‐level characteristics of participants with T1DM were associated with variability in the observed effect sizes for LV GLS [15]. Univariable random‐effects meta‐regression models were fitted using study‐level mean values of age, diabetes duration and HbA1c, where available [20]. Meta‐regression analyses were restricted to studies reporting numerical mean values for the relevant covariates. Studies lacking numerical reporting of baseline characteristics were included in the primary meta‐analysis but excluded from exploratory meta‐regression analyses. Multivariable meta‐regression models were not performed due to the risk of overfitting, potential collinearity among study‐level covariates, and the inherent limitations of ecological analyses [20, 21]. All meta‐regression analyses were considered exploratory and hypothesis‐generating [15, 22].

### 2.6 Certainty of Evidence

The overall certainty of evidence for LV GLS was evaluated using the Grading of Recommendations, Assessment, Development and Evaluation (GRADE) methodology [23]. Evidence from each study was assessed across five domains: risk of bias, inconsistency, indirectness, imprecision, and publication bias. The certainty of the evidence was classified as high when there was strong confidence that the true effect closely matched the estimated effect. Evidence was rated as moderate if the true effect was likely to be close to the estimate, low when confidence in the estimate was limited, and very low when there was minimal confidence that the estimate reflected the true effect. Observational studies initially start as low‐certainty evidence but could be upgraded if, for example, a large magnitude of effect or a clear dose‐response gradient was observed. Conversely, evidence could be downgraded for serious limitations in any of the domains.

## 3 Results

### 3.1 Search Results and Study Characteristics

**FIGURE 1:**
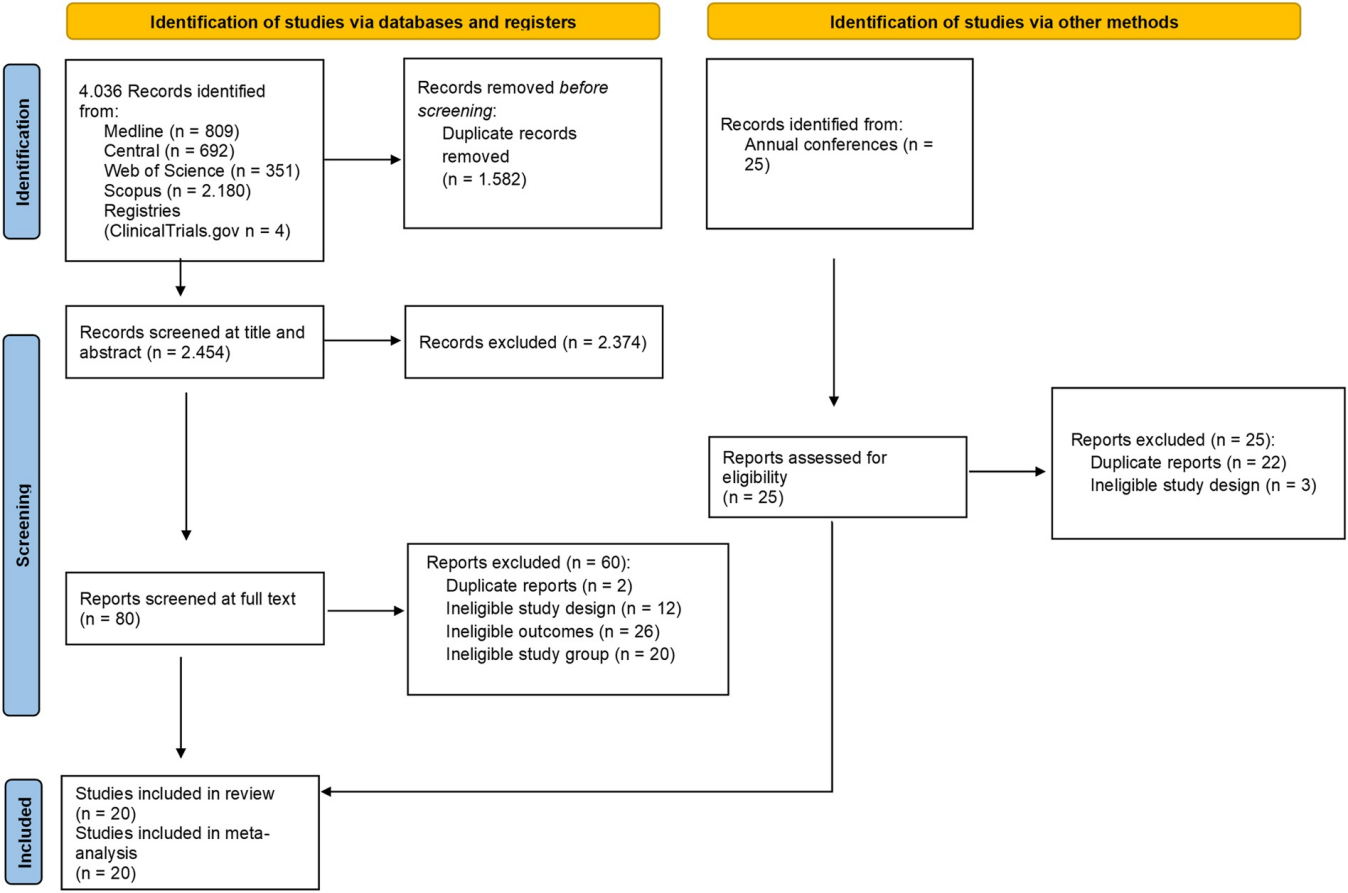
Preferred reporting items for systematic reviews and meta‐analyses (PRISMA) flow chart for the identification inclusion and exclusion of studies.

The systematic literature search identified a total of 4032 records across all databases. After removal of duplicates, 2454 records remained and were screened based on titles and abstracts. Of these, 80 articles were retrieved for full‐text assessment. Following full‐text evaluation, 20 studies met the predefined inclusion criteria and were included in the meta‐analysis. The flow diagram of the study selection process is shown in Figure 1.

The included studies were published between 2014 and 2025 and comprised a total of 1188 children and adolescents with T1DM and 1028 healthy controls. This total overall encompasses all eligible participants. Note that the primary 2D analysis incorporates 1086 T1DM patients and 935 controls across 18 studies, as two studies contributed exclusively to the 3D‐STE analysis [6, 24] and one study provided both 2D and 3D data [25]. Inclusion and exclusion criteria of each study are presented in Supporting Information S1: Table S2. Study designs were predominantly observational, mainly cross‐sectional case–control studies, while a smaller number provided baseline data from cohort studies. Although our predefined eligibility criteria allowed for the inclusion of studies utilising extractable normative data, all studies finally included in the quantitative synthesis provided data for a concurrent, matched healthy control group. Consequently, no reference‐range data were utilised in the meta‐analysis and all pooled estimates represent direct pairwise comparisons between T1DM patients and concurrent controls. Across studies, LV GLS was assessed using 2D speckle‐tracking echocardiography. Two studies reported LV GLS using exclusively 3D speckle‐tracking echocardiography [6, 24], while one study reported GLS using both 2D and 3D techniques [25]. Study populations covered a wide paediatric age range, from early childhood to late adolescence, and included both newly diagnosed and long‐standing diabetes cases. A summary of the baseline characteristics of the included studies is presented in Table 1. Baseline characteristics were extracted and presented as originally described by the study authors.

**TABLE 1:** Baseline characteristics of the included studies (data are presented as mean ± SD, unless otherwise reported in the original studies).

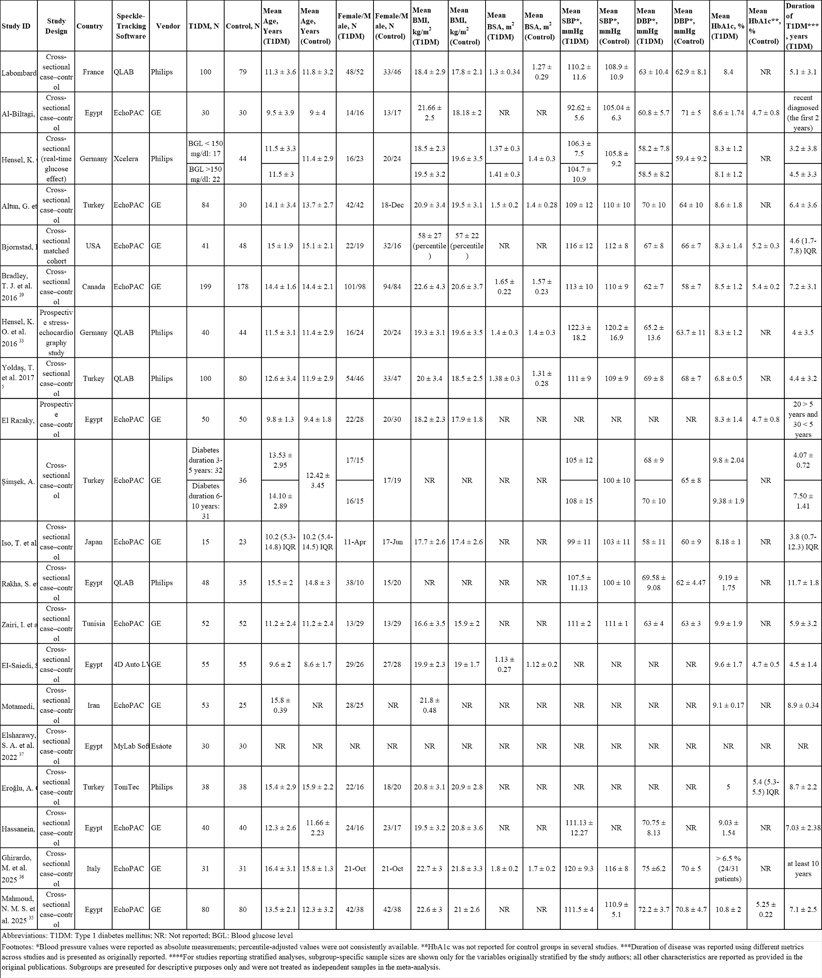

Ultimately, no RCTs met the inclusion criteria for quantitative synthesis; all included studies were observational and risk of bias was assessed independently for all studies using the ROBINS‐I V2 tool (Supporting Information S1: Table S3–S5). Most observational studies were judged to be at a moderate risk of bias, mainly due to residual confounding and participant selection [5, 6, 24, 25, 26, 27, 28, 29, 30, 32, 33, 34, 36, 37, 39, 40, 41]. Confounding was the most frequent source of bias, reflecting incomplete adjustment for prespecified baseline covariates, while one study was deemed to be at serious risk of bias because of missing baseline confounder data and limited assessment of group comparability [38]. Bias related to exposure classification, missing data and outcome measurement was generally low, whereas bias in selection of the reported result was commonly moderate, owing to the absence of prespecified analysis plans and the reporting of multiple echocardiographic outcomes. No study was judged to be at critical risk of bias and two studies were rated as low risk [31, 35]. Studies at serious risk of bias were retained in the primary analysis and examined in sensitivity analyses.

### 3.2 Global Longitudinal Strain

#### 3.2.1 Overall and Primary 2D LV GLS Analysis

**FIGURE 2:**
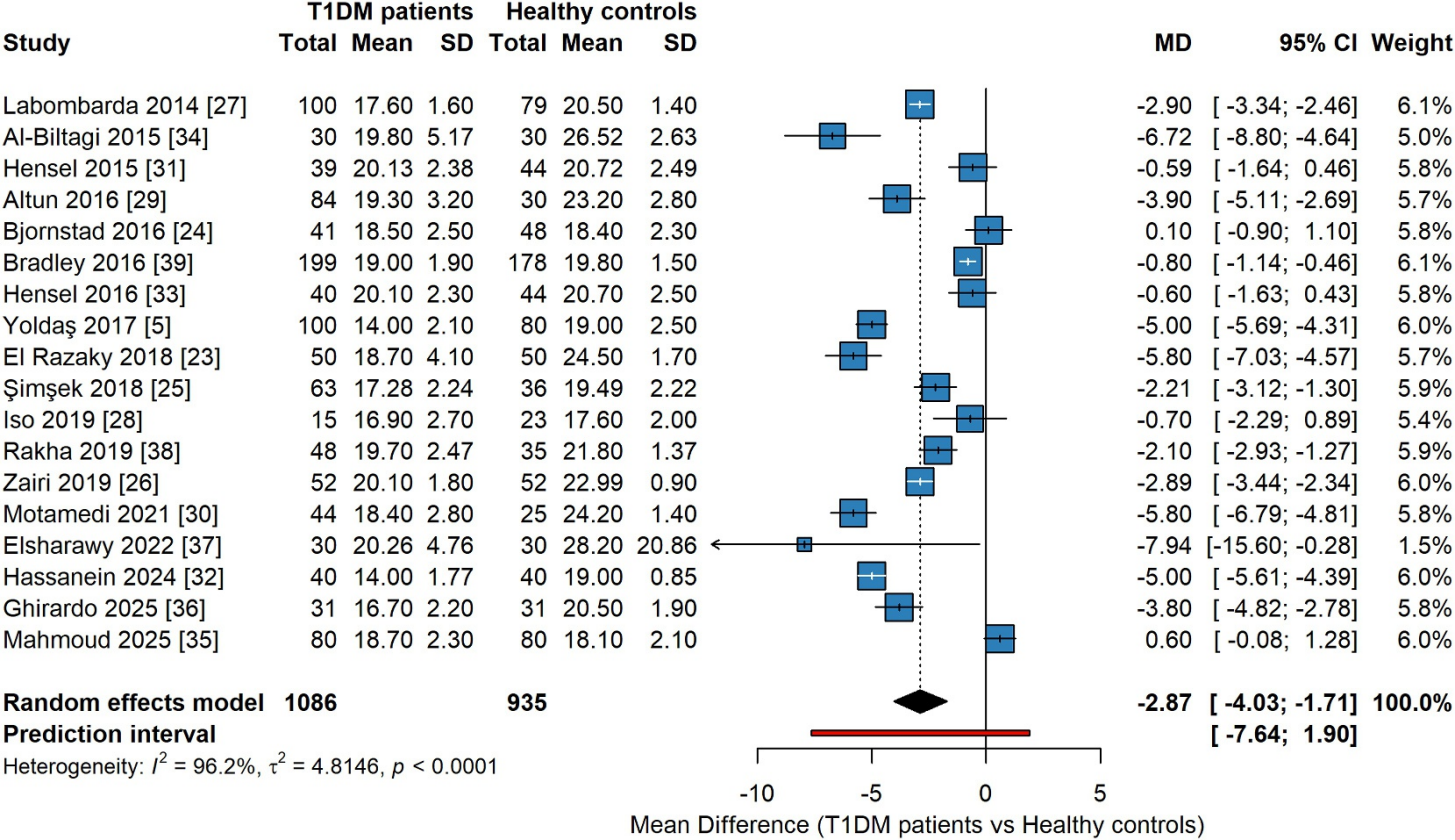
Forest plot of 2D left ventricular global longitudinal strain (LV GLS, %) in paediatric population with T1DM versus healthy controls. Overall effect.

An initial overall combined meta‐analysis incorporating all eligible studies (both 2D and 3D modalities) was performed. The pooled all‐modality estimate demonstrated a significant reduction in LV GLS among children and adolescents with T1DM compared with healthy controls (SMD −1.16, 95% CI −1.64 to −0.68, *p* < 0.001), confirming global longitudinal impairment across different echocardiographic techniques (Supporting Information S1: Figure S1). Focusing on the primary modality, a total of 18 studies contributed data to the specific meta‐analysis of 2D LV GLS, comprising 1086 children and adolescents with T1DM and 935 healthy controls. Compared with healthy controls, children with T1DM demonstrated a significantly impaired 2D LV GLS (MD −2.87%, 95% CI −4.03 to −1.71), indicating less negative strain values and therefore reduced myocardial deformation (Figure 2). A random‐effects model was applied due to substantial between‐study heterogeneity, which was confirmed by a high degree of inconsistency (I^2^ = 96.2%, *p* < 0.0001). Despite the observed heterogeneity, the direction of the effect was largely consistent across studies, with the majority reporting worse 2D LV GLS in the T1DM group. The prediction interval ranged from −7.64% to 1.90%, suggesting that while the average effect indicates impaired myocardial deformation, the magnitude of LV GLS differences may vary across different clinical and methodological settings. Egger's regression test did not indicate evidence of small‐study effects (*t* = −1.13, *p* = 0.28). Visual inspection of the funnel plot did not suggest marked asymmetry (Supporting Information S1: Figure S2).

According to the GRADE framework, the overall certainty of evidence for the association between T1DM and impaired LV GLS in children and adolescents was rated as very low (Supporting Information S1: Table S6). The body of evidence was derived exclusively from observational studies (initially starting at low certainty), and the certainty was downgraded by one additional level due to serious inconsistency (moderate‐to‐substantial heterogeneity). Consequently, while the direction of the effect suggests impaired myocardial deformation in T1DM, confidence in the exact magnitude of the pooled estimate is limited by clinical and methodological variability.

#### 3.2.2 Exploratory Analysis of 3D LV GLS

Three studies provided data for an exploratory meta‐analysis of 3D LV GLS, including 143 children with T1DM and 143 healthy controls [6, 24, 25]. In the pooled random‐effects analysis, no statistically significant difference in 3D LV GLS was observed between children with T1DM and controls (MD −3.22%, 95% CI −11.64 to 5.20, Supporting Information S1: Figure S3). Between‐study heterogeneity was substantial (I^2^ = 97.6%, *p* < 0.0001), and the prediction interval was wide (−19.94% to 13.51%), indicating marked uncertainty regarding the magnitude and direction of the effect.

#### 3.2.3 Vendor‐Based Subgroup Analysis

**FIGURE 3:**
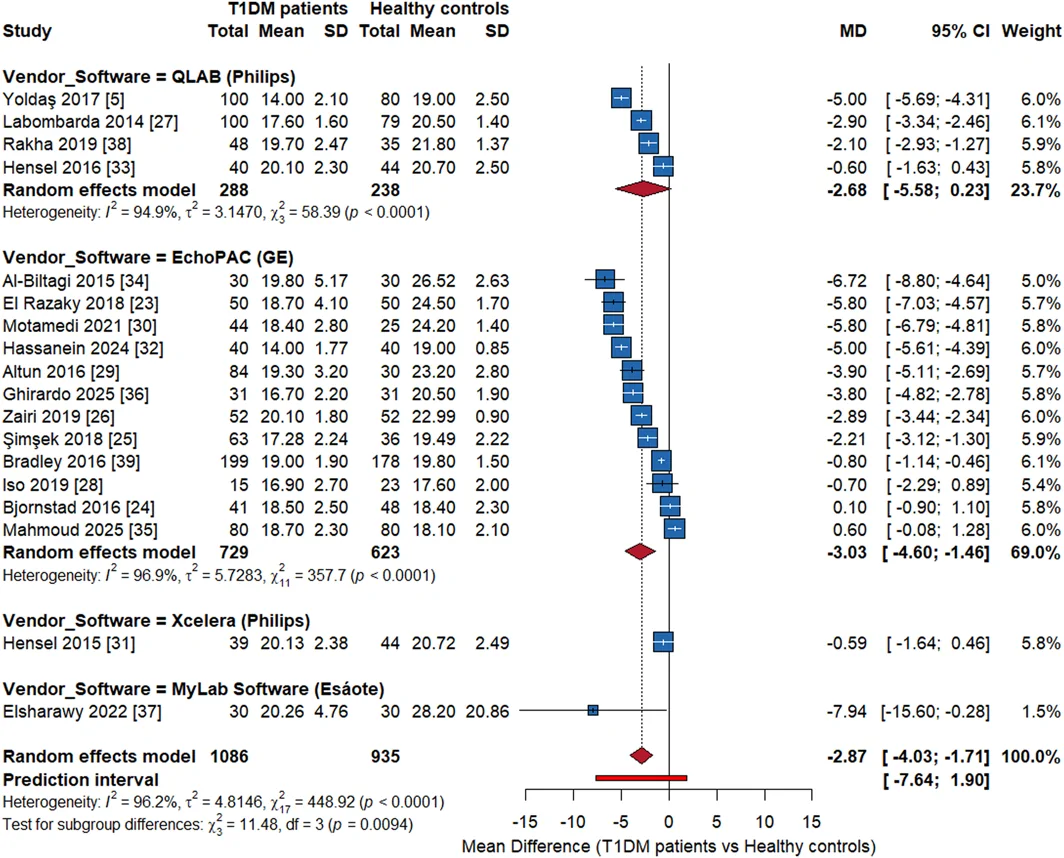
Forest plot of 2D left ventricular global longitudinal strain (LV GLS, %) in paediatric population with T1DM versus healthy controls. Subgroup analysis based on vendor‐specific software.

A prespecified subgroup analysis was conducted according to the vendor‐specific software used for GLS analysis (Figure 3). In studies using EchoPAC (GE), children with T1DM exhibited a significantly impaired LV GLS compared with controls (MD −3.03%, 95% CI −4.60 to −1.46), based on 729 T1DM patients and 623 controls. In studies using QLAB (Philips), the pooled estimate did not demonstrate a statistically significant difference between groups (MD −2.68%, 95% CI −5.58 to 0.23; 288 T1DM patients and 238 controls). Single‐study subgroups were available for Xcelera (Philips) (MD −0.59%, 95% CI −1.64 to 0.46) and MyLab (Esaote) (MD −7.94%, 95% CI −15.60 to −0.28), precluding robust within‐subgroup pooling. A statistically significant difference between subgroups was observed (*p* for subgroup differences = 0.0094), indicating that the estimated magnitude of LV GLS impairment varied according to the software platform used. Substantial heterogeneity persisted within major subgroups, particularly among EchoPAC‐based studies.

#### 3.2.4 Sensitivity Analysis

A sensitivity analysis excluding one study assessed as being at serious risk of bias [38], yielded results consistent with the primary analysis (Supporting Information S1: Figure S4). Following exclusion, 17 studies remained, including 1056 children with T1DM and 905 healthy controls, and the pooled estimate continued to demonstrate a significantly impaired 2D LV GLS in the T1DM group (MD −2.80%, 95% CI −3.96 to −1.63). Between‐study heterogeneity remained substantial (I^2^ = 96.4%, *p* < 0.0001), and the prediction interval (−7.58% to 1.98%) overlapped with that of the main analysis.

### 3.3 Meta‐Regression

Exploratory meta‐regression analyses were performed to assess whether selected study‐level characteristics were associated with variability in 2D LV GLS effect estimates. Univariable random‐effects meta‐regression did not demonstrate a significant association between study‐level mean age and the magnitude of 2D LV GLS difference between children and adolescents with T1DM and healthy controls (*β* = 0.03 per year, 95% CI −0.58 to 0.64, *p* = 0.92, Table 2). Visual inspection of the corresponding bubble plot revealed substantial between‐study variability within similar age ranges, suggesting that age alone did not account for the observed heterogeneity (Supporting Information S1: Figure S5). Similarly, no statistically significant association was observed between mean duration of T1DM and LV GLS differences (*β* = −0.22 per year, 95% CI −0.82 to 0.38, *p* = 0.43, Table 2 and Supporting Information S1: Figure S6). Finally, mean HbA1c was not significantly associated with the magnitude of LV GLS impairment (*β* = 0.50 per 1% increase in HbA1c, 95% CI −0.93 to 1.94, *p* = 0.46, Table 2 and Supporting Information S1: Figure S7).

**TABLE 2:** Meta‐regression analyses.

Moderator	Beta	SE	Z	*p*_value	CI_lower	CI_upper
Age (years)						
Mean age (years)	0.03	0.29	0.1	0.92	−0.58	0.64
HbA1c (%)						
Mean HbA1c (%)	0.50	0.66	0.76	0.46	−0.93	1.94
T1DM duration (years)						
Mean duration (years)	−0.22	0.27	−0.81	0.43	−0.82	0.38

## 4 Discussion

In this meta‐analysis, we assessed differences in LV GLS between children and adolescents with T1DM and healthy controls as a marker of subclinical myocardial dysfunction. Our initial combined synthesis across all echocardiographic modalities demonstrated a significant overall impairment in LV longitudinal strain. Focusing on the primary analysis, we confirmed a significant reduction in 2D LV GLS in youths with T1DM, supporting the presence of early myocardial involvement even in the absence of overt cardiac disease. Importantly, these alterations were observed in populations largely characterised by preserved conventional systolic indices, underscoring the superior sensitivity of GLS for detecting subtle myocardial dysfunction at an early stage of the disease. The wide prediction interval underscores that while impaired LV GLS is a consistent finding, its magnitude may vary substantially across clinical settings. Although substantial heterogeneity was observed, no evidence of publication bias was detected in the primary analysis based on funnel plot inspection and Egger's regression test.

Similar reductions in GLS have been described across paediatric cohorts with varying disease duration and metabolic control, supporting the concept of early subclinical myocardial involvement [30]. However, not all previous studies have reported significant differences in LV GLS between paediatric T1DM patients and controls [26, 32]. Variability in study populations, disease duration, glycaemic control and echocardiographic methodology may partly explain these discrepancies [42]. The observed reduction in LV GLS likely reflects early alterations in myocardial function related to chronic hyperglycaemia, insulin deficiency and metabolic dysregulation. Proposed mechanisms include impaired myocardial energetics, microvascular dysfunction, low‐grade inflammation, oxidative stress and early interstitial fibrosis, which preferentially affect longitudinally oriented subendocardial fibres [18]. These pathophysiological processes may precede detectable changes in LV EF, rendering GLS particularly sensitive for identifying early myocardial injury in paediatric T1DM. A preceding meta‐analysis by Gupta et al. reported lower LV GLS values in asymptomatic children and adolescents with T1DM compared with healthy controls, supporting the presence of early subclinical myocardial dysfunction despite preserved ejection fraction [12]. That analysis focused exclusively on 2D speckle‐tracking echocardiography and primarily quantified pooled strain values across studies [12].

In contrast to the consistent impairment observed with 2D GLS, the exploratory analysis of 3D GLS did not demonstrate a statistically significant difference between children with T1DM and healthy controls. This finding should be interpreted with caution, as it was based on a very limited number of studies and was characterised by extreme between‐study heterogeneity. The discrepancy between 2D and 3D GLS findings is expected. Although 3D GLS is theoretically advantageous by avoiding out‐of‐plane motion, it has not yet achieved comparable reproducibility or clinical validation in paediatric populations [43]. Lower temporal and spatial resolution, greater dependency on image quality, and limited standardisation across vendors and software platforms likely contribute to the observed variability [44]. In line with our findings, previous paediatric studies assessing 3D GLS have reported inconsistent results, with some demonstrating impaired strain and others demonstrating preserved myocardial deformation [25, 45]. These discrepancies likely reflect the methodological limitations of 3D speckle‐tracking in children rather than the true absence of myocardial involvement [43]. Consequently, within a research context, current evidence indicates that 2D LV GLS is a sensitive echocardiographic parameter for detecting early subclinical myocardial alterations in paediatric T1DM, whereas the clinical utility of 3D GLS remains to be established [19].

Subgroup analysis based on vendor‐specific software demonstrated variability in the magnitude of LV GLS impairment across platforms, providing complementary insights to previous meta‐regression approaches that incorporated individual software packages as covariates. Studies employing EchoPAC software consistently demonstrated impaired LV GLS in children with T1DM, whereas results derived from QLAB‐based analyses were more heterogeneous and did not reach statistical significance at the pooled level. These findings should not be interpreted as evidence of preserved myocardial deformation with specific software platforms, nor as systematic overestimation of dysfunction by others. Rather, they likely reflect well‐recognized inter‐vendor variability in strain algorithms, tracking methods, and post‐processing pipelines [46, 47]. Despite these differences, the overall pooled effect across all software platforms remained statistically significant and directionally consistent, indicating that software‐related variability primarily affects the magnitude rather than the direction of the observed effect. Furthermore, the exclusion of one study with serious risk of bias did not materially alter the magnitude or direction of the pooled effect, indicating that the observed association between T1DM and impaired LV GLS was not driven by lower‐quality data. Notably, between‐study heterogeneity remained substantial, suggesting that variability is more likely driven by underlying clinical differences and methodological factors, including echocardiographic protocols and vendor‐specific strain software, rather than by risk of bias alone. Finally, visual inspection of the chronologically ordered forest plot for 2D LV GLS did not reveal a clear temporal trend in the magnitude of impairment over the past decade, suggesting that the observed differences are a persistent clinical finding rather than a strict artefact of evolving echocardiographic technologies.

Exploratory meta‐regression analyses did not identify significant associations between LV GLS differences and study‐level mean age, diabetes duration, or HbA1c. The absence of an age‐related effect suggests that observed GLS differences are not primarily driven by variation in age across studies, although this finding should be interpreted cautiously given the ecological nature of meta‐regression [20, 21]. Similarly, although a trend toward greater impairment with longer disease duration was observed, this association did not reach statistical significance. No association was observed between study‐level mean HbA1c and LV GLS differences, a finding that may reflect the limited ability of aggregate HbA1c values to capture individual or cumulative glycaemic exposure relevant to myocardial remodelling. This interpretation aligns with evidence from adult cohorts indicating that single or averaged HbA1c measurements may inadequately reflect the cumulative metabolic burden most relevant to myocardial deformation, particularly in cross‐sectional analyses [48]. Furthermore, during childhood and adolescence, myocardial mechanics are profoundly influenced by rapid somatic growth and pubertal physiological changes [19, 30]. It is highly plausible that in this specific developmental window, aggregate HbA1c has limited direct biological relevance to myocardial strain, providing a distinct hypothesis for the lack of association observed in our meta‐regression. Long‐term observational data further support the concept that cumulative glycaemic exposure and glycaemic variability, rather than contemporaneous HbA1c alone, are more closely related to cardiovascular structural and functional alterations [49, 50]. The preceding meta‐analysis suggested a possible but inconsistent association between HbA1c and LV GLS in paediatric T1DM, with reported effects that were weak and not consistently statistically significant across included studies [12].

The high degree of heterogeneity observed across our analyses persisted even after software‐based subgroup and sensitivity analyses. This residual variability strongly suggests the influence of unmeasured or inconsistently reported clinical confounders across the included studies. Factors such as pubertal stage, detailed adiposity metrics (e.g., BMI z‐scores), variations in insulin regimens or delivery methods and the undetected coexistence of early microvascular complications likely account for a substantial portion of this heterogeneity [9, 30, 31]. While the overall direction of impaired myocardial deformation was largely consistent, indicating subclinical cardiac involvement, the exact magnitude of this effect is highly variable among different clinical phenotypes.

Several limitations should be acknowledged. All GLS data were derived from observational studies and residual confounding cannot be excluded. Vendor‐ and software‐specific influences on GLS values may have contributed to heterogeneity and subgroup analyses were conducted at the study level, limiting causal inference. The exploratory 3D GLS findings are constrained by the small number of available studies, limited sample sizes, substantial heterogeneity and lack of standardisation in acquisition and analysis. Although random‐effects modelling and prediction intervals were employed to account for between‐study variability, pooled estimates should be interpreted as average effects rather than precise individual‐level measures. In addition, most included studies were cross‐sectional, limiting the ability to assess the prognostic significance of reduced LV GLS or its temporal progression over time. Moreover, substantial clinical heterogeneity existed with respect to disease stage at the time of assessment, as paediatric T1DM populations ranged from newly diagnosed patients to children and adolescents with long‐standing disease receiving established insulin therapy. Although comparisons were consistently performed against healthy control groups within each study, differences in disease duration and treatment exposure may have contributed to between‐study variability in LV GLS estimates and could not be fully accounted for using study‐level meta‐regression. Furthermore, inconsistent reporting and adjustment for disease‐related confounders across studies limited a more detailed exploration of effect modification at the individual participant level. Finally, the inability to extract and adjust for an extended list of potential confounders‐such as pubertal development stage and specific insulin regimens‐prevented a richer exploration of the observed heterogeneity.

Taken together, the results of this meta‐analysis support the presence of early subclinical myocardial dysfunction in children and adolescents with T1DM and suggest that 2D LV GLS (but not currently 3D GLS) may serve as a sensitive imaging parameter in a research setting. Given the very low certainty of evidence and substantial heterogeneity, routine clinical screening cannot be recommended at this stage. However, subclinical myocardial dysfunction detected during childhood represents the earliest stage of diabetic cardiomyopathy, which may progressively lead to overt adult heart failure if metabolic disturbances persist. Establishing a definitive prognostic link would have significant clinical implications. For instance, the identification of early 2D LV GLS impairment in high‐risk phenotypes—such as those with prolonged disease duration or consistently suboptimal glycaemic control—could eventually trigger closer cardiovascular surveillance. This could prompt earlier, intensified interventions, including strict glycaemic management and targeted lifestyle modifications, long before traditional ejection fraction declines.

In conclusion, the identification of impaired 2D LV GLS in asymptomatic paediatric patients with T1DM suggests that myocardial involvement begins early in life. Future longitudinal studies with harmonised echocardiographic protocols are essential to determine whether these early 2D STE abnormalities definitively predict future clinical cardiac dysfunction and to establish evidence‐based algorithms for targeted surveillance and early preventive strategies.

## Author Contributions

M.K. and A.C. conceived the study, which was designed by A.S., G.S. and E.E‐F. A.S. and E.E‐F. reviewed and selected articles, and A.C. resolved conflicts. A.S. and E.E‐F. extracted data. A.S. and G.S. assessed the risk of bias, and A.C. resolved conflicts. A.S., G.S. and E.E‐F. carried out the statistical analysis. All authors contributed to the interpretation of the data. A.S. wrote the first manuscript draft, which A.C., M.K., and N.P. critically revised for important intellectual content. All authors approved the final version of the manuscript.

## Funding

The authors have nothing to report.

## Conflicts of Interest

The authors declare no conflicts of interest.

## Supporting information


Supporting Information S1

## Data Availability

The data that support the findings of this study are available from the corresponding author upon reasonable request.
