# Supplementary material for: Left Ventricular Global Longitudinal Strain as a Marker of Subclinical Myocardial Dysfunction in Youths With Type 1 Diabetes Mellitus: An Updated Systematic Review and Meta‐Analysis
Source: Diabetes Metab Res Rev. 2026 Aug 25;42(6):e70220. doi: 10.1002/dmrr.70220 (PMC13505227; doi:10.1002/dmrr.70220)
Supplement: Supplementary file 1 [file DMRR-42-e70220-s001.docx]

**Left ventricular global longitudinal strain as a marker of subclinical myocardial dysfunction in youths with Type 1 diabetes mellitus: A systematic review and meta-analysis**

**Authors**

Athina Stamati^1^, Georgia Sotiriou^2^, Elpida Emmanoulidou-Fotoulaki^2^, Nikoleta Printza^2^, Maria Kavga^3^, Athanasios Christoforidis^2^

**Author affiliations**

^1^ School of Medicine, Faculty of Health Science, Aristotle University of Thessaloniki, Thessaloniki, Greece

^2^ 1^st^ Paediatric Department, School of Medicine, Faculty of Health Sciences, Aristotle University of Thessaloniki, Ippokratio General Hospital, Thessaloniki, Greece

^3^ 3^rd^ Paediatric Department, School of Medicine, Faculty of Health Sciences, Aristotle University of Thessaloniki, Ippokratio General Hospital, Thessaloniki, Greece

**Table of contents**

[**Table S1 – Search strategies**	4](#_Toc236382705)

[**Table S2 – Inclusion and exclusion criteria of each study included**	6](#_Toc236382706)

[**Table S3 – ROBINS-I v2 assessment for the primary outcome (LV GLS)**	9](#_Toc236382707)

[**Table S4 – Prespecified baseline confounders for the assessment of bias due to confounding (ROBINS-I v2)**	14](#_Toc236382708)

[**Table S5 – Detailed ROBINS-I v2 assessment with domain-specific key justifications for the primary outcome (LV GLS)**	15](#_Toc236382709)

[**Table S6 – GRADE assessment**	28](#_Toc236382710)

[**Figure S1 – Forest plot of combined 2D and 3D Left ventricular global longitudinal strain (LV GLS, %) in paediatric population with T1DM versus healthy controls. Overall effect.**	30](#_Toc236382711)

[**Figure S2 – Funnel plot for LV GLS**	31](#_Toc236382712)

[**Figure S3 – Forest plot of 3D Left ventricular global longitudinal strain (LV GLS, %) in paediatric population with T1DM versus healthy controls. Overall effect.**	32](#_Toc236382713)

[**Figure S4 - Meta-analysis results after excluding studies with serious risk of bias**	33](#_Toc236382714)

[**Figure S5 – Bubble plot of meta-regression between LV GLS difference and mean age**	34](#_Toc236382715)

[**Figure S6 – Bubble plot of meta-regression between LV GLS difference and mean duration of T1DM**	35](#_Toc236382716)

[**Figure S7 – Bubble plot of meta-regression between LV GLS difference and mean HbA1c**	36](#_Toc236382717)

# **Table S1 – Search strategies**

**PUBMED – Search strategy - 809**

#1 ("diabetes mellitus, type 1"[MeSH Terms] OR "type 1 diabetes mellitus"[All Fields] OR "type 1 diabetes"[All Fields] OR "insulin depend*"[All Fields] OR "T1DM"[All Fields])

#2 ("echo*"[All Fields] OR "myocardi*"[All Fields] OR (("longitudinal"[All Fields] OR "longitudinally"[All Fields]) AND ("sprains and strains"[MeSH Terms] OR ("sprains"[All Fields] AND "strains"[All Fields]) OR "sprains and strains"[All Fields] OR "strain"[All Fields] OR "strains"[All Fields] OR "strain s"[All Fields])) OR ("doppler"[All Fields] OR "doppler s"[All Fields] OR "dopplers"[All Fields]) OR "GLS"[All Fields] OR (("myocardially"[All Fields] OR "myocardium"[MeSH Terms] OR "myocardium"[All Fields] OR "myocardial"[All Fields]) AND ("dysfunctional"[All Fields] OR "dysfunctionals"[All Fields] OR "dysfunctioning"[All Fields] OR "dysfunctions"[All Fields] OR "physiopathology"[MeSH Subheading] OR "physiopathology"[All Fields] OR "dysfunction"[All Fields])) OR (("cardiacs"[All Fields] OR "heart"[MeSH Terms] OR "heart"[All Fields] OR "cardiac"[All Fields]) AND ("diagnostic imaging"[MeSH Subheading] OR ("diagnostic"[All Fields] AND "imaging"[All Fields]) OR "diagnostic imaging"[All Fields] OR "ultrasound"[All Fields] OR "ultrasonography"[MeSH Terms] OR "ultrasonography"[All Fields] OR "ultrasonics"[MeSH Terms] OR "ultrasonics"[All Fields] OR "ultrasounds"[All Fields] OR "ultrasound s"[All Fields])))

#3 ("child*"[All Fields] OR ("paediatrics"[All Fields] OR "pediatrics"[MeSH Terms] OR "pediatrics"[All Fields] OR "paediatric"[All Fields] OR "pediatric"[All Fields]) OR "adolescen*"[All Fields])

#4 #1 AND #2 AND #3

**COCHRANE – Search strategy - 692**

#1	(type 1 diabetes OR insulin-depend* OR T1DM)	99109

#2	(echo* OR myocardi* OR "longitudinal strain" OR doppler OR GLS OR "myocardial dysfunction" OR "cardiac ultrasound")	94006

#3	(child* OR paediatric OR adolescen*)	373220

#4	#1 AND #2 AND #3	692

**SCOPUS – Search strategy - 2.180**

TITLE-ABS-KEY(("type 1 diabetes" OR insulin-depend* OR T1DM)

AND (echo* OR myocardi* OR "longitudinal strain" OR doppler OR GLS OR "myocardial dysfunction" OR "cardiac ultrasound")

AND (child* OR paediatric OR adolescen*))

**WEB OF SCIENCE Core Collection – Search strategy - 351**

TS=(("type 1 diabetes" OR insulin-depend* OR T1DM)

AND (echo* OR myocardi* OR "longitudinal strain" OR doppler OR GLS OR "myocardial dysfunction" OR "cardiac ultrasound")

AND (child* OR paediatric OR adolescen*))

# **Table S2 – Inclusion and exclusion criteria of each study included**

| **Study ID** | **Study Design** | **Study population** | **Inclusion criteria** | **Exclusion criteria** |
| --- | --- | --- | --- | --- |
| Labombarda, F. et al. 2014 ^27^ | Cross-sectional case–control | Children with T1DM patients and healthy controls comparable to age, gender, heart rate and blood pressure (2012–2013) | Age 5–18 years, T1DM ≥ 1 year, insulin therapy; controls with normal echocardiography | Cardiopathy, significant comorbidity, cardioactive medications, hypertension, dyslipidaemia, obesity, smoking, or recent T1DM diagnosis |
| Al-Biltagi, M. A. et al. 2015 ^34^ | Cross-sectional case–control | Newly diagnosed paediatric T1DM patients and healthy age- and sex-matched controls (2012-2013) | Newly diagnosed T1DM, early evaluation within 48 hours of diagnosis | Cardiovascular disease, hypertension, metabolic or systemic conditions affecting cardiac function, other diabetes types, or use of cardiovascular medications |
| Hensel, K. O. et al. 2015 ^31^ **** | Cross-sectional (real-time glucose effect) | Asymptomatic, normotensive children with uncomplicated T1DM and healthy age- and sex-matched controls | Paediatric T1DM without clinical cardiovascular disease | Diabetes-related end-organ damage, cardiovascular-affecting comorbidities, or use of cardioactive medications (except insulin) |
| Altun, G. et al. 2016 [29] | Cross-sectional case–control | Children with T1DM patients and healthy age-, sex- and body mass index matched controls | Paediatric T1DM; availability of echocardiographic and 2D-STE data | Not reported |
| Bjornstad, P. et al. 2016 ^24^ | Cross-sectional matched cohort | Observational study within the RESISTANT cohort in pubertal adolescents with T1DM and healthy controls similar in age, body mass index, Tanner stage, level of habitual physical activity, and sex | Age 12–19 years, pubertal stage, sedentary lifestyle; T1DM per ADA criteria with autoantibodies; healthy controls with normal OGTT | Hypertension, anaemia, renal dysfunction, poor glycaemic control, smoking, pregnancy, cardio-/metabolically active medications, or conditions limiting exercise testing. Family history of T1DM in control group |
| Bradley, T. J. et al. 2016 ^39^ | Cross-sectional case–control | Children with T1DM and healthy age-, sex- and height matched controls; Observational study based on baseline data from the non-interventional arm of the AdDIT cohort | Adolescents with T1DM | Participation in interventional AdDIT trial, vasoactive medications, or significant cardiac, renal, metabolic, or systemic disease |
| Hensel, K. O. et al. 2016 ^33^ | Prospective stress-echocardiography study | Children with T1DM and healthy age- and sex-matched controls | Age 6–17 years, T1DM, good general health; healthy controls with normal cardiovascular evaluation | Cardiovascular-affecting comorbidities, inflammatory disease, proteinuria, cardioactive or systemic medications (except insulin), severe obesity, abnormal ECG or exercise test findings, or evidence of end-organ damage |
| Yoldaş, T. et al. 2017 ^5^ | Cross-sectional case–control | Children with T1DM and healthy age- and sex-matched controls (2014-2015) | Age 5–18 years, T1DM > 1 year, insulin therapy, LVEF > 60%, good metabolic control | Cardiac disease, hypertension, diabetic complications, or cardioactive medications |
| El Razaky, O. et al. 2018 ^23^ | Prospective case–control | Children with T1DM patients and healthy age-, sex- and weight matched controls (2020-2021) | Age <18 years, T1DM | Cardiac disease, infection, type 2 diabetes, diabetic nephropathy, or systemic disease affecting cardiac function |
| Şimşek, A. et al. 2018 ^25^ **** | Cross-sectional case–control | Children with T1DM and healthy age-matched controls | Age 7–18 years, T1DM on insulin therapy, absence of cardiac symptoms; controls with normal physical and echocardiographic findings | Microvascular complications or other systemic diseases |
| Iso, T. et al. 2019 ^28^ | Cross-sectional case–control | Petients with T1DM and healthy age- and sex-matched controls divided into three age groups | Age 5–40 years, T1DM, availability of LV strain analysis; controls with normal ECG and echocardiography | Cardiovascular disease, hypertension, cardiac-affecting comorbidities, or use of cardioprotective drugs |
| Rakha, S. et al. 2019 ^38^ | Cross-sectional case–control | Children with long-standing T1DM and healthy age- and sex-matched controls (2015-2016) | Age ≤ 18 years, T1DM duration ≥ 10 years | Chronic comorbid illness, structural or rhythm cardiac disease, cardiomyopathy, congenital heart disease, or poor image quality |
| Zairi, I. et al. 2019 ^26^ | Cross-sectional case–control | Children with T1DM patients and healthy age- and sex-matched controls (2014-2016) | Age 5–15 years, T1DM > 1 year, insulin therapy, LVEF > 55%, normal ECG, no congenital heart disease; controls with normal echocardiography | Incomplete echocardiographic data or insufficient apical views precluding assessment |
| EI-Saiedi, S. A. et al. 2020 ^6^ | Cross-sectional case–control | Paediatric patients with T1DM and healthy age- and sex-matched controls (2018-2019) | Children with T1DM | Arrhythmia, structural heart disease, obesity affecting image quality, or diabetes-related comorbidities |
| Motamedi, M. et al. 2021 ^30^ | Cross-sectional case–control | Children with T1DM and healthy age-matched controls (2018-2019) | Age < 18 years, T1DM duration > 5 years, availability of laboratory evaluation and echocardiographic assessment including 2D echocardiography and 2D speckle-tracking echocardiography. | Not reported |
| Elsharawy, S. A. et al. 2022 ^37^ | Cross-sectional case–control | Children with T1DM and comparable to age and gender healthy controls (2019-2021) | T1DM, age < 18 years, LVEF > 55%, absence of congenital heart disease | Structural or rheumatic heart disease, type 2 diabetes, heart failure or cardiomyopathy, chronic kidney disease, hypertension, prior cardiac surgery, family history of hypercholesterolaemia. Control participants had no history of diabetes, congenital heart disease, hypertension, or dyslipidaemia. |
| Eroğlu, A. G. et al. 2023 ^22^ | Cross-sectional case–control | Children with T1DM patients and healthy age-, sex- and body measurments matched controls (2019) | Age < 21 years, T1DM duration ≥ 5 years | Non-T1DM chronic disease, cardiac disease, T1DM complications, obesity, hypertension, non-insulin medications, or poor echo image quality |
| Hassanein, S. A. et al. 2024 ^32^ | Cross-sectional case–control | Children with T1DM patients and healthy age- and sex-matched controls (2020-2021) | Age 7–18 years, T1DM duration ≥ 5 years; controls with normal cardiac structure and function | Arrhythmia, congenital heart disease, systemic disease affecting cardiac function, or use of cardioactive medications |
| Ghirardo, M. et al. 2025 ^36^ | Cross-sectional case–control | Adolescents with T1DM patients and healthy age- and sex-matched controls (2023) | Age 13–19 years, T1DM duration ≥ 10 years, intensive therapy; healthy controls with normal ECG and echocardiography | Non-T1DM diabetes, cardiovascular or genetic heart disease, missing glycaemic data, poor acoustic window, minor cardiac defects, rhythm abnormalities, or poor image quality |
| Mahmoud, N. M. S. et al. 2025 ^35^ | Cross-sectional case–control | Children with T1DM patients and healthy age- and sex-matched controls (2023-2025) | Age 6–18 years, T1DM duration > 5 years, insulin therapy; healthy controls free of chronic disease | Systemic or endocrine disease (other than T1DM), congenital heart disease, acute infection, non-insulin medications, or additional cardiovascular risk factors |
| Abbreviations: Type 1 diabetes mellitus, T1DM; Oral Glucose Tolerance Test, OGTT | | | | |

# **Table S3 – ROBINS-I v2 assessment for the primary outcome (LV GLS)**

| **Study ID** | **Estimand** | **Confounding (D1)** | **Classification of interventions (D2)** | **Selection of participants into the study (D3)** | **Missing data (D4)** | **Measurement of the outcome (D5)** | **Selection of the reported result (D6)** | **Overall** | **Key justification** |
| --- | --- | --- | --- | --- | --- | --- | --- | --- | --- |
| Labombarda, F. et al. 2014 ^27^ | ITT | Moderate | Low | Moderate | Low | Low | Moderate | Moderate | Unadjusted GLS comparison with residual confounding (duration/HbA1c); post-hoc subgroup analyses without prespecified SAP. |
| Al-Biltagi, M. A. et al. 2015 ^34^ | ITT | Moderate | Low | Moderate | Low | Low | Moderate | Moderate | Unadjusted strain comparisons with major baseline differences (BMI, BP, HR, HbA1c); multiple outcomes without prespecified SAP. |
| Hensel, K. O. et al. 2015 ^31^ **** | ITT | Moderate | Low | Moderate | Low | Low | Moderate | Moderate | Acute glycaemic stratification without multivariable adjustment; multiple strain outcomes without prespecified SAP. |
| Altun, G. et al. 2016 [29] | ITT | Moderate | Low | Moderate | Low | Low | Moderate | Moderate | Residual confounding (duration, HbA1c, BP) and lack of prespecified analysis plan. |
| Bjornstad, P. et al. 2016 ^24^ | ITT | Moderate | Low | Moderate | Low | Low | Moderate | Moderate | Incomplete control for diabetes duration and glycaemic control. |
| Bradley, T. J. et al. 2016 ^39^ | ITT | Low | Low | Low | Low | Low | Low | Low | Comprehensive multivariable adjustment and prespecified analyses; minimal residual bias. |
| Hensel, K. O. et al. 2016 ^33^ | ITT | Moderate | Low | Moderate | Low | Low | Moderate | Moderate | High-quality blinded STE, but unadjusted primary strain comparisons with subgroup-based confounding control only. |
| Yoldaş, T. et al. 2017 ^5^ | ITT | Moderate | Low | Moderate | Low | Low | Moderate | Moderate | Residual confounding (duration/HbA1c) despite good metabolic control restriction; no prespecified SAP. |
| El Razaky, O. et al. 2018 ^23^ | ITT | Moderate | Low | Moderate | Low | Low | Moderate | Moderate | Residual confounding (duration/HbA1c) and multiple strain analyses without prespecified SAP. |
| Şimşek, A. et al. 2018 ^25^ **** | ITT | Moderate | Low | Moderate | Low | Low | Moderate | Moderate | Unadjusted GLS comparison with subgroup analyses by duration/HbA1c; multiple outcomes without prespecified SAP. |
| Iso, T. et al. 2019 ^28^ | ITT | Moderate | Low | Moderate | Low | Low | Moderate | Moderate | Residual confounding by diabetes duration; multiple layer-specific strain analyses without prespecified SAP. |
| Rakha, S. et al. 2019 ^38^ | ITT | Low | Low | Low | Low | Low | Low | Low | Adequate multivariable adjustment for GLS; HbA1c identified as independent predictor. |
| Zairi, I. et al. 2019 ^26^ | ITT | Moderate | Low | Moderate | Low | Low | Moderate | Moderate | Unadjusted GLS despite strong correlations with duration and HbA1c; multiple analyses without prespecified SAP. |
| EI-Saiedi, S. A. et al. 2020 ^6^ | ITT | Moderate | Low | Moderate | Low | Low | Moderate | Moderate | Unadjusted GLS with biomarker-driven analyses; multiple endpoints without prespecified SAP. |
| Motamedi, M. et al. 2021 ^30^ | ITT | Moderate | Low | Moderate | Low | Low | Moderate | Moderate | Unadjusted GLS comparison with extensive subgroup/segmental analyses and no prespecified SAP. |
| Elsharawy, S. A. et al. 2022 ^37^ | ITT | Serious | Low | Moderate | Low | Low | Moderate | Serious | Absence of baseline confounder data precludes assessment of residual confounding. |
| Eroğlu, A. G. et al. 2023 ^22^ | ITT | Moderate | Low | Moderate | Low | Low | Moderate | Moderate | Residual confounding (duration/HbA1c) and multiple strain analyses without prespecified SAP. |
| Hassanein, S. A. et al. 2024 ^32^ | ITT | Moderate | Low | Moderate | Low | Low | Moderate | Moderate | Unadjusted GLS comparisons despite wide variability in HbA1c and BP; multiple echo outcomes without prespecified SAP. |
| Ghirardo, M. et al. 2025 ^36^ | ITT | Moderate | Low | Moderate | Low | Low | Moderate | Moderate | Residual confounding (duration, glycaemic control, BP/BSA) and use of external control cohort. |
| Mahmoud, N. M. S. et al. 2025 ^35^ | ITT | Moderate | Low | Moderate | Low | Low | Moderate | Moderate | Unadjusted GLS analysis despite regression for other outcomes; multiple echo parameters without prespecified SAP. |
| Abbreviations: Intention-to-treat, ITT; Statistical analysis plan, SAP | | | | | | | | | |

# **Table S4 – Prespecified baseline confounders for the assessment of bias due to confounding (ROBINS-I v2)**

| **Confounder (pre-specified)** | **Rationale** | **Measured** | **Adjusted** | **Expected bias** |
| --- | --- | --- | --- | --- |
| Age | LV GLS varies with age and myocardial maturation in children and adolescents | Yes | Frequently | Direction depends on age imbalance between groups |
| Sex | Sex-related differences in myocardial deformation and cardiac geometry | Yes | Frequently | Depends on sex distribution between groups |
| Diabetes duration | Cumulative exposure to hyperglycaemia and metabolic stress affects myocardial mechanics | Yes | Rarely | Overestimation of GLS impairment |
| HbA1c | Chronic hyperglycaemia is associated with myocardial dysfunction and microvascular damage | Yes | Rarely | Overestimation of GLS impairment |

# **Table S5 – Detailed ROBINS-I v2 assessment with domain-specific key justifications for the primary outcome (LV GLS)**

| **Study ID** | **Confounding (D1)** | **Classification of interventions (D2)** | **Selection of participants into the study (D3)** | **Missing data (D4)** | **Measurement of the outcome (D5)** | **Selection of the reported result (D6)** | **Overall** |
| --- | --- | --- | --- | --- | --- | --- | --- |
| Labombarda, F. et al. 2014 ^27^ | Groups were comparable for age, sex, heart rate and blood pressure; however, no multivariable adjustment was performed for diabetes duration or HbA1c in the primary GLS comparison, leaving residual baseline confounding. | T1DM defined by WHO criteria with permanent insulin requirement; controls rigorously screened and free of cardiometabolic disease; exposure status determined prior to echocardiography. | Single-centre convenience sample; controls recruited from children evaluated for innocent murmurs, potentially limiting representativeness though not selected by outcome. | GLS successfully measured in the vast majority of segments; excluded segments due to poor tracking were few and non-differential, with no evidence of group-specific missingness. | GLS assessed using standardized 2D speckle-tracking with predefined acquisition protocols and reported intra- and inter-observer reproducibility. | No prespecified statistical analysis plan; multiple strain parameters and post-hoc subgroup analyses (HbA1c tertiles) reported, allowing potential selective emphasis. | **Moderate**; Overall judgement driven by residual confounding and lack of prespecified analyses; no domain judged at serious or critical risk. |
| Al-Biltagi, M. A. et al. 2015 ^34^ | Age- and sex-matched controls were used; however, there was no multivariable adjustment for major baseline confounders (BMI, blood pressure, heart rate, glycaemic status), several of which differed significantly between groups, leaving material residual confounding. | Type 1 diabetes clearly defined and assessed at diagnosis; controls were healthy children without systemic or cardiac disease; exposure status ascertained prior to echocardiography. | Single-centre convenience sample of recently diagnosed T1DM; controls recruited from hospital population, potentially limiting representativeness though not selected based on echocardiographic outcomes. | Complete echocardiographic and laboratory data reported for all enrolled participants; no evidence of differential missingness. | Ventricular strain assessed using standardized 2D speckle-tracking and RT3DE with offline analysis; same equipment and operator used, with objective outcome measurement. | No prespecified statistical analysis plan; multiple ventricular outcomes (LV and RV strain, MPI, EF, TAPSE) and extensive correlation analyses with resistin increase risk of selective emphasis. | **Moderate**; Overall judgement driven by substantial residual confounding and lack of prespecified analyses; no domain at serious or critical risk. |
| Hensel, K. O. et al. 2015 ^31^ **** | Age- and sex-matched controls with similar anthropometrics, BP and diabetes duration; however, no multivariable adjustment was performed for baseline confounders (e.g., heart rate, BMI, HbA1c) in the primary GLS comparisons, leaving residual confounding, particularly given acute glycaemic stratification. | T1DM clearly defined; controls rigorously screened; real-time blood glucose measured contemporaneously with echocardiography, ensuring accurate exposure classification. | Single-centre convenience sample of uncomplicated T1DM; controls recruited from healthy volunteers, potentially limiting representativeness though not selected based on echocardiographic outcomes. | Complete echocardiographic and laboratory data reported for all analysed participants; no evidence of differential missingness. | GLS and strain rate assessed with standardized 2D speckle-tracking using blinded offline analysis and excellent inter-observer reproducibility (<6%). | No prespecified statistical analysis plan; multiple deformation parameters (CS, LS, strain rate) and subgroup analyses by glucose level reported, allowing potential selective emphasis. | **Moderate**; Overall judgement driven by residual baseline confounding and lack of prespecified analyses; outcome measurement quality was high with no serious or critical domains. |
| Altun, G. et al. 2016 [29] | Matching for age, sex and BMI was performed; however, no multivariable adjustment for diabetes duration, blood pressure or HbA1c was applied in the primary T1DM–control comparison, resulting in residual baseline confounding. | Clear definition of T1DM and healthy controls; exposure status ascertained prior to echocardiographic assessment, with minimal risk of misclassification. | Single-center convenience sample with restrictive inclusion criteria; controls recruited separately, limiting representativeness but without selection based on outcome. | Complete echocardiographic strain data available for all included participants; no indication of differential missingness. | LV GLS assessed using standardized 2D speckle-tracking echocardiography with the same equipment, software and single experienced operator for all participants. | No prespecified statistical analysis plan reported; multiple strain parameters and subgroup analyses performed, allowing potential selective emphasis of significant results. | **Moderate**; Overall judgement driven by residual confounding and potential selective reporting; no domain judged at serious or critical risk. |
| Bjornstad, P. et al. 2016 ^24^ | Matching by design for age, sex, BMI, Tanner stage and physical activity; no adjustment for diabetes duration or HbA1c in the primary T1DM–control comparison, leaving residual baseline confounding | Clear definition of T1DM (ADA criteria and autoantibody positivity) and confirmation of normoglycaemia in controls with OGTT; minimal risk of exposure misclassification. | Convenience sample restricted to pubertal, sedentary adolescents (RESISTANT cohort), limiting representativeness but without selection based on outcome. | Echocardiographic strain data available for all included participants; no evidence of differential missingness between groups. | LV GLS assessed using standardized 2D speckle-tracking echocardiography with the same equipment, software and experienced operator for all participants. | No prespecified analysis plan reported; multiple strain parameters assessed, with potential for selective emphasis of results. | **Moderate**; Overall judgement driven by residual confounding and potential selective reporting; no domains at serious or critical risk. |
| Bradley, T. J. et al. 2016 ^39^ | Extensive adjustment for major baseline confounders (age, sex, BSA, SBP/DBP, HbA1c, BMI, lipids, ACR) with multivariable models; residual confounding unlikely to materially affect GLS estimates. | Clear definition of T1DM; controls screened for absence of cardiometabolic disease and vasoactive medications; negligible exposure misclassification. | Large, well-characterized cohorts recruited independently of outcomes; controls contemporaneously recruited with explicit eligibility criteria; minimal selection related to GLS. | Complete echocardiographic data in both groups; no evidence of differential missingness affecting GLS. | GLS measured with standardized 2D speckle-tracking protocols, validated software, and quality control; objective outcome with low risk of differential measurement. | Prespecified analytical framework with correction for multiple testing; GLS reported alongside comprehensive deformation measures, reducing selective reporting risk. | **Low**; All domains judged at low risk; robust adjustment strategy and large sample size support low overall risk of bias for GLS. |
| Hensel, K. O. et al. 2016 ^33^ | Age- and sex-matched controls with similar anthropometrics and blood pressure; however, no multivariable adjustment for diabetes duration or HbA1c was applied in the primary strain comparisons, with duration and glycaemic control analysed only in subgroup analyses, leaving residual baseline confounding. | Type 1 diabetes clearly defined; controls rigorously screened and free of cardiovascular disease; exposure status ascertained prior to echocardiographic assessment. | Single-centre convenience sample of consecutive T1DM patients; controls recruited from healthy volunteers, potentially limiting representativeness though not selected based on echocardiographic outcomes. | Complete strain and strain-rate data reported for analysed participants; exclusions due to predefined criteria and image quality were minimal and non-differential. | LV strain and strain rate assessed with standardized 2D speckle-tracking using blinded offline analysis by two independent readers and excellent interobserver reproducibility (<6%). | No prespecified statistical analysis plan; multiple deformation parameters (strain and strain rate at rest and stress) and subgroup analyses reported, allowing potential selective emphasis. | **Moderate**; Overall judgement driven by residual confounding and lack of prespecified analyses; outcome measurement quality was high with no serious or critical domains. |
| Yoldaş, T. et al. 2017 ^5^ | Matching for age and sex with similar BP; however, no multivariable adjustment for diabetes duration or HbA1c (restricted range by inclusion criteria), leaving residual baseline confounding. | Clear WHO-based definition of T1DM; controls rigorously screened for cardiac disease; exposure status ascertained prior to echocardiography. | Single-center convenience sample; controls recruited from referrals for murmurs/chest pain, potentially limiting representativeness though not selected by outcome. | Complete GLS data reported for included participants; no evidence of differential missingness between groups. | GLS measured with standardized 2D speckle-tracking using the same equipment/software and a single experienced operator; objective outcome with low risk of differential measurement. | No prespecified analysis plan; multiple strain directions and subgroup/correlation analyses reported, allowing potential selective emphasis. | **Moderate**; Overall judgement driven by residual confounding and potential selective reporting; no domain at serious or critical risk. |
| El Razaky, O. et al. 2018 ^23^ | Matching for age, sex and weight; however, no multivariable adjustment for diabetes duration or HbA1c (analyzed only in correlations), leaving residual baseline confounding. | Clear definition of type 1 DM; controls selected as healthy children without cardiac disease; exposure status ascertained prior to echocardiography. | Single-center convenience sample; controls recruited from outpatient clinics for minor complaints, potentially limiting representativeness though not selected by outcome. | Complete strain data reported for all included participants; no evidence of differential missingness. | GLS assessed with standardized 2D- and 3D-STE using validated software, consistent acquisition protocols and excellent interobserver reliability (ICCs >0.9). | No prespecified analysis plan; multiple strain modalities (2D and 3D) and laboratory correlations examined, allowing potential selective emphasis. | Overall judgement driven by residual confounding and potential selective reporting; no domain at serious or critical risk. |
| Şimşek, A. et al. 2018 ^25^ **** | Although age-, sex-, BMI- and blood pressure–matched controls were used, no multivariable adjustment was applied for key baseline confounders such as diabetes duration and HbA1c in the primary GLS comparison, leaving residual baseline confounding. | T1DM defined according to standard criteria with exclusion of microvascular complications; controls were healthy children with normal echocardiography; exposure status was clearly ascertained prior to outcome assessment. | Single-centre convenience sample; controls recruited from children referred for murmur or chest pain, potentially limiting representativeness though not selected based on echocardiographic outcomes. | Complete echocardiographic and laboratory data reported for all included participants; no dropouts or differential missingness described. | LV GLS assessed using standardized 2D speckle-tracking echocardiography with the same equipment and software, and objective outcome measurement. | No prespecified statistical analysis plan; multiple echocardiographic outcomes (GLS, circumferential and radial strain, diastolic indices) and extensive correlation analyses reported, allowing potential selective emphasis. | **Moderate**; Overall judgement driven by residual confounding and lack of prespecified analyses; no domain judged at serious or critical risk. |
| Iso, T. et al. 2019 ^28^ | Age- and sex-matching was performed and major cardiac comorbidities were excluded; however, no multivariable adjustment for diabetes duration or HbA1c was applied in the primary group comparisons, leaving residual baseline confounding. | T1DM clearly defined; controls rigorously screened and free of cardiovascular disease and cardioprotective medication; exposure status ascertained prior to echocardiography. | Single-centre convenience sample; controls recruited from healthy volunteers and children evaluated for innocent murmurs, potentially limiting representativeness though not selected by outcome. | Complete echocardiographic strain data available for all included participants; no evidence of differential missingness. | Layer-specific LS measured with standardized 2D speckle-tracking, blinded offline analysis by two observers, and excellent intra- and inter-observer reproducibility. | No prespecified statistical analysis plan; multiple strain parameters (layer-specific LS and CS across levels) and extensive correlation analyses reported, allowing potential selective emphasis. | **Moderate**; Overall judgement driven by residual confounding (notably diabetes duration) and lack of prespecified analyses; no domain at serious or critical risk. |
| Rakha, S. et al. 2019 ^38^ | Age- and sex-matched controls with comparable anthropometrics and blood pressure; multivariable regression performed for GLS including HbA1c, diabetes duration, BMI, BP and other clinical variables, with HbA1c identified as the only independent predictor. | T1DM defined according to ADA criteria with ≥10-year duration; controls healthy and rigorously screened; exposure status ascertained prior to echocardiography. | Prospective recruitment of consecutive paediatric patients with long-standing T1DM and age- and sex-matched controls; no selection based on echocardiographic outcomes. | Complete GLS data available for all analysed participants; exclusions due to image quality were predefined and non-differential. | LV GLS assessed with standardized 2D speckle-tracking by a single experienced operator using validated software. | GLS reported as a primary outcome with consistent analytical approach; multivariable modelling reduces risk of selective emphasis despite multiple strain parameters. | **Low**; All ROBINS-I v2 domains judged at low risk; confounding adequately controlled for the GLS outcome with no evidence of selection or measurement bias. |
| Zairi, I. et al. 2019 ^26^ | Age- and sex-matching performed with similar blood pressure and BMI; however, no multivariable adjustment for diabetes duration or HbA1c was applied in the primary GLS comparison, leaving residual baseline confounding. | T1DM defined according to WHO criteria with permanent insulin requirement; controls rigorously screened and free of cardiometabolic disease; exposure status determined prior to echocardiography. | Single-centre convenience sample; controls recruited from children evaluated for innocent murmurs, potentially limiting representativeness though not selected by outcome. | Complete LV GLS data reported for all included participants; exclusions due to image quality were limited and non-differential. | GLS assessed using standardized 2D speckle-tracking with blinded offline analysis by two observers and excellent intra- and inter-observer reproducibility (ICC ≥0.90). | No prespecified statistical analysis plan; multiple strain outcomes (LV GLS, RV FWLS) and extensive correlation and ROC analyses performed, allowing potential selective emphasis. | **Moderate**; Overall judgement driven by residual confounding and lack of prespecified analyses; no domain judged at serious or critical risk. |
| EI-Saiedi, S. A. et al. 2020 ^6^ | Age- and sex-matching with similar anthropometrics; however, no multivariable adjustment for diabetes duration or HbA1c was applied for LV GLS, with biomarker-focused analyses leaving residual baseline confounding. | T1DM clearly defined; controls age- and sex-matched and free of cardiac disease; exposure status ascertained prior to echocardiography. | Single-centre convenience sample with exclusions for comorbidities; controls recruited from hospital population, potentially limiting representativeness though not selected by outcome. | Complete 3D-STE GLS data reported for analysed participants; no evidence of differential missingness. | LV GLS assessed using standardized real-time 3D speckle-tracking with validated software and offline analysis; objective outcome with low risk of differential measurement. | No prespecified statistical analysis plan; multiple echocardiographic endpoints and extensive biomarker comparisons (NRI/IDI/AUC) increase risk of selective emphasis. | **Moderate**; Overall judgement driven by residual confounding and lack of prespecified analyses; no domain judged at serious or critical risk. |
| Motamedi, M. et al. 2021 ^30^ | No multivariable adjustment was applied for major baseline confounders (diabetes duration, HbA1c, blood pressure, BMI); comparisons relied on unadjusted group contrasts and subgroup/correlation analyses, leaving residual baseline confounding. | Type 1 diabetes clearly defined with disease duration ≥5 years; controls were age-matched healthy children with normal echocardiography; exposure status ascertained prior to STE. | Single-centre convenience sample; only 44/53 diabetic patients had valid STE due to image quality exclusions, and controls were drawn from an existing laboratory cohort, potentially limiting representativeness. | xclusions due to poor image quality were predefined; GLS data were complete for all analysed participants with no evidence of differential missingness between groups. | LV GLS measured using standardized 2D speckle-tracking echocardiography with the same vendor and acquisition protocol for patients and controls; objective outcome measurement. | No prespecified statistical analysis plan; extensive segmental analyses, multiple subgroup comparisons and correlations were performed, allowing potential selective emphasis. | **Moderate**; Overall judgement driven by residual confounding and lack of prespecified analyses; outcome measurement quality was adequate with no serious or critical domains. |
| Elsharawy, S. A. et al. 2022 ^37^ | Important baseline confounders (diabetes duration, glycaemic control, blood pressure and BMI) were not reported or adjusted for; absence of baseline comparability data precludes assessment of residual confounding, likely leading to substantial distortion of the GLS effect estimate. | Type 1 diabetes clearly defined by standard criteria; controls were healthy and screened; exposure status ascertained prior to echocardiography. | Single-centre convenience sample; controls recruited from children evaluated for murmurs with normal echocardiography, potentially limiting representativeness though not selected by outcome. | Complete GLS data reported for all analysed participants; exclusions were predefined with no evidence of differential missingness. | LV GLS measured using standardized 2D speckle-tracking with offline analysis by a single observer blinded to clinical data; objective outcome with low risk of differential measurement. | No prespecified statistical analysis plan; multiple global and segmental strain outcomes and correlation analyses reported, allowing potential selective emphasis. | **Serious**; Overall judgement driven by serious risk of bias due to unmeasured and unreported baseline confounding; other domains did not reach serious or critical risk. |
| Eroğlu, A. G. et al. 2023 ^22^ | Matching for age, sex and body size with exclusion of obesity and hypertension; however, no multivariable adjustment for diabetes duration or HbA1c (assessed only in correlation analyses), resulting in residual baseline confounding. | Type 1 diabetes defined according to ADA criteria; controls were healthy children without cardiac disease; exposure status determined before echocardiographic assessment. | Single-centre convenience sample of adolescents with ≥5-year T1DM; controls recruited from the same institution, limiting representativeness but without selection based on outcome. | Participants with inadequate image quality were excluded a priori; complete GLS data available for all analysed participants with no evidence of differential missingness. | GLS assessed using standardized 3D and speckle-tracking echocardiography with blinded offline analysis and excellent intra- and inter-observer reproducibility. | No prespecified statistical analysis plan; multiple deformation parameters (2D/3D strain, twist, torsion) evaluated, allowing potential selective emphasis of findings. | **Moderate**; Overall judgement driven by residual confounding and lack of prespecified analyses; no domain judged at serious or critical risk. |
| Hassanein, S. A. et al. 2024 ^32^ | Although age- and sex-matching was performed and major cardiac comorbidities were excluded, no multivariable adjustment was applied for important baseline confounders (diabetes duration, HbA1c, blood pressure, lipid profile), which showed wide variability among cases, leaving residual baseline confounding. | Type 1 diabetes defined according to ADA criteria with duration ≥5 years; controls were healthy children with structurally normal hearts; exposure status was clearly ascertained prior to echocardiographic assessment. | Single-centre convenience sample from a tertiary diabetes clinic; controls recruited from hospital-based population, potentially limiting representativeness though not selected based on echocardiographic outcomes. | Complete echocardiographic and laboratory data reported for all 80 participants; no dropouts or differential missingness described. | LV and RV GLS measured using standardized 2D speckle-tracking echocardiography (EchoPAC, GE) with predefined acquisition protocols and objective outcome assessment. | No prespecified statistical analysis plan; multiple echocardiographic outcomes (LV/RV GLS, EF, FS, TDI indices) and extensive correlation analyses were conducted, allowing potential selective emphasis. | **Moderate**; Overall judgement driven by residual confounding and lack of prespecified analyses; outcome measurement quality was adequate with no serious or critical domains. |
| Ghirardo, M. et al. 2025 ^36^ | Age- and sex-matching performed; no multivariable adjustment for diabetes duration, HbA1c/TIR, blood pressure or BSA, leaving residual baseline confounding. | Type 1 diabetes defined by ISPAD criteria; controls defined as healthy with normal ECG/echo; minimal risk of exposure misclassification. | Single-center convenience sample of adolescents with ≥10-year T1DM; controls drawn from a previous laboratory cohort, limiting representativeness and comparability. | Complete GLS data reported for included participants; no evidence of differential missingness. | GLS measured with standardized 2D speckle tracking; single experienced operator and blinded offline analysis using the same equipment/software. | No prespecified analysis plan; multiple echocardiographic and myocardial work outcomes assessed, allowing potential selective emphasis. | **Moderate**; Overall judgement driven by residual confounding and potential selective reporting; no domain at serious or critical risk. |
| Mahmoud, N. M. S. et al. 2025 ^35^ | Age- and sex-matching was performed; although multivariable regression was used for vascular outcomes (cIMT), no adjusted analysis was applied for LV GLS, and diabetes duration, HbA1c and BMI differed between groups, leaving residual baseline confounding. | T1DM defined according to ADA criteria; controls rigorously screened and free of chronic disease; exposure status ascertained prior to echocardiography. | Single-centre convenience sample including both inpatients and outpatients; controls recruited from siblings, hospital visitors and community volunteers, potentially limiting representativeness. | Complete echocardiographic and laboratory data reported for all 160 participants; no dropouts or differential missingness. | LV GLS assessed using standardized speckle-tracking echocardiography with the same equipment and blinded, experienced operators; objective outcome with low risk of differential measurement. | No prespecified statistical analysis plan; multiple echocardiographic outcomes (GLS from different views, TAPSE, MPI, E/e′) and extensive correlation/regression analyses reported, allowing potential selective emphasis. | **Moderate**; Overall judgement driven by residual confounding for GLS and lack of prespecified analyses; no domain at serious or critical risk. |

# **Table S6 – GRADE assessment**

| **Quality assesment** | | | | | | | **Summary of findings** | | | |  |
| --- | --- | --- | --- | --- | --- | --- | --- | --- | --- | --- | --- |
|  | | | | | | | **No of patient** | | **Effects** | **Quality** | **Comments** |
| **No of studies** | **Study design** | **Risk of Bias** | **Incosistency** | **Indirectness** | **Imprecision** | **Other considerations** | **Type 1 diabetes mellitus** | **Healthy controls** | **Absolute (95% CI)** |  |  |
| **Percentage of Global longitudinal strain (GLS)** | | | | | | | | | | |  |
| 18 | observational | not serious | serious | not serious | not serious | none | 1.086 | 935 | MD -2.87% lower (-4.03 to -1.71) | ⊕ Very Low | Consistent reduction in LV GLS among children and adolescents with T1DM compared with healthy controls. Certainty downgraded due to heterogeneity. |

# **Figure S1 – Forest plot of combined 2D and 3D Left ventricular global longitudinal strain (LV GLS, %) in paediatric population with T1DM versus healthy controls. Overall effect.**

# **Figure S2 – Funnel plot for LV GLS**

# **Figure S3 – Forest plot of 3D Left ventricular global longitudinal strain (LV GLS, %) in paediatric population with T1DM versus healthy controls. Overall effect.**

# **Figure S4 - Meta-analysis results after excluding studies with serious risk of bias**

# **Figure S5 – Bubble plot of meta-regression between LV GLS difference and mean age**

# **Figure S6 – Bubble plot of meta-regression between LV GLS difference and mean duration of T1DM**

# **Figure S7 – Bubble plot of meta-regression between LV GLS difference and mean HbA1c**
